# Therapeutic Potential of Cranberry Proanthocyanidins in Addressing the Pathophysiology of Metabolic Syndrome: A Scrutiny of Select Mechanisms of Action

**DOI:** 10.3390/antiox14030268

**Published:** 2025-02-26

**Authors:** Francis Feldman, Mireille Koudoufio, Alain Théophile Sané, Valérie Marcil, Mathilde Foisy Sauvé, James Butcher, Natalie Patey, Catherine Martel, Schohraya Spahis, Haonan Duan, Daniel Figeys, Yves Desjardins, Alain Stintzi, Emile Levy

**Affiliations:** 1 Azraeli Research Centre, Sainte-Justine University Health Centre, Montreal, QC H3T 1C5, Canada; francis.feldman@umontreal.ca (F.F.); mireille.koudoufio@umontreal.ca (M.K.); sanealaintheo@gmail.com (A.T.S.); valerie.marcil@umontreal.ca (V.M.); mathilde.foisy.sauve@umontreal.ca (M.F.S.); schohraya.spahis.hsj@ssss.gouv.qc.ca (S.S.); 2 Department of Nutrition, Université de Montréal, Montreal, QC H3T 1A8, Canada; 3 School of Pharmaceutical Sciences, Ottawa Institute of Systems Biology, University of Ottawa, Ottawa, ON K1H 1M5, Canada; jbutcher@uottawa.ca (J.B.); hduan084@uottawa.ca (H.D.); dfigeys@uottawa.ca (D.F.); astintzi@uottawa.ca (A.S.); 4 Department of Biochemistry, Microbiology and Immunology, University of Ottawa, Ottawa, ON K1H 8M5, Canada; 5 Department of Pathology and Cell Biology, Université de Montréal, Montreal, QC H3C 3J7, Canada; natalie.patey.med@ssss.gouv.qc.ca; 6 Montreal Heart Institute Research Centre, Montreal, QC H1T 1C8, Canada; catherine.martel.9@umontreal.ca; 7 Departement of Medicine, Faculty of Medicine, Université de Montréal, Montreal, QC H3T 1J4, Canada; 8 Department of Biochemistry & Molecular Medicine, Université de Montréal, Montreal, QC H3C 3J7, Canada; 9 Institute of Nutrition and Functional Foods, Laval University, Quebec, QC G1V 4L3, Canada; yves.desjardins@fsaa.ulaval.ca

**Keywords:** obesity, dyslipidemia, inflammation, hepatic steatosis, gut–liver axis, intestinal microbiota

## Abstract

Metabolic syndrome (MetS) constitutes a spectrum of interconnected conditions comprising obesity, dyslipidemia, hypertension, and insulin resistance (IR). While a singular, all-encompassing treatment for MetS remains elusive, an integrative approach involving tailored lifestyle modifications and emerging functional food therapies holds promise in preventing its multifaceted manifestations. Our main objective was to scrutinize the efficacy of cranberry proanthocyanidins (PAC, 200 mg/kg/day for 12 weeks) in mitigating MetS pathophysiology in male mice subjected to standard Chow or high-fat/high-fructose (HFHF) diets while unravelling intricate mechanisms. The administration of PAC, in conjunction with an HFHF diet, significantly averted obesity, evidenced by reductions in body weight, adiposity across various fat depots, and adipocyte hypertrophy. Similarly, PAC prevented HFHF-induced hyperglycemia and hyperinsulinemia while also lessening IR. Furthermore, PAC proved effective in alleviating key risk factors associated with cardiovascular diseases by diminishing plasma saturated fatty acids, as well as levels of triglycerides, cholesterol, and non-HDL-C levels. The rise in adiponectin and drop in circulating levels of inflammatory markers showcased PAC’s protective role against inflammation. To better clarify the mechanisms behind PAC actions, gut–liver axis parameters were examined, showing significant enhancements in gut microbiota composition, microbiota-derived metabolites, and marked reductions in intestinal and hepatic inflammation, liver steatosis, and key biomarkers associated with endoplasmic reticulum (ER) stress and lipid metabolism. This study enhances our understanding of the complex mechanisms underlying the development of MetS and provides valuable insights into how PAC may alleviate cardiometabolic dysfunction in HFHF mice.

## 1. Introduction

Nutrition has a critical impact on human and animal health at every stage of life [1]. More specifically, functional foods are increasingly recognized as highly influential nutrients from the gestational period to old age [2,3]. Among these bioactive molecules, polyphenols are gaining an incrementally favourable standing, giving their potential to prevent or at least lower the threats of non-communicable diseases [4,5]. These compounds are now recognized as prebiotics that interacts reciprocally with the microbiota: they can modulate its composition and are also degraded by it, releasing potentially bioactive metabolites [6,7].

Recently, we have reported the beneficial impact of proanthocyanidins (PACs), known as natural flavan-3-ols oligomers elaborated by various fruits, including cranberries [8,9,10,11,12]. In the well-established human enterocyte Caco-2/15 cell line, PAC displayed the ability to prevent lipid peroxidation, avert induction of the inflammatory process, reverse lipogenesis in favour of fatty acid (FA) β-oxidation, downregulate gluconeogenesis, and enhance insulin sensitivity [13]. Similarly, in an obesogenic mouse model, PAC protected against obesity, insulin resistance (IR), oxidative stress (OxS), inflammation, lipid deposition in the liver via upregulation in FA β-oxidation, and prevented microbiota dysbiosis [8,9,10,11,12,14]. Despite these remarkable advances, the underlying mechanisms explaining the potential of PAC across the gut–liver axis and its associated dyslipidemias remain poorly documented. Due to their high degree of polymerization and de facto absent bioavailability, the study of PAC presents the singular opportunity to isolate the gut and its role along the gut–liver axis in the development of non-communicable diseases.

Unravelling the intricate web of physiological, molecular, and systemic aberrations that underpin metabolic syndrome (MetS) can serve to reveal key pathways and biomarkers associated with its development and progression. Furthermore, the detection of mechanisms is paramount for devising effective prevention and treatment strategies. As no singular pharmaceutical intervention currently exists that comprehensively targets all the underlying factors simultaneously associated with MetS, numerous endeavours must be invested in to uncover a unified treatment for all its conditions, including excess body fat, IR-mediated metabolic glucose abnormalities, dyslipidemia and high blood pressure [15].

With the aim of advancing our understanding, we have expanded upon our previous study [14] by incorporating PAC to explore additional mechanisms underlying their actions. Our objectives were multifaceted: firstly, we sought to validate and build upon prior findings regarding the favourable repercussions of PAC on obesity and IR [16,17]. Secondly, we conducted a comprehensive analysis of multiple inflammatory biomarkers, recognizing their pivotal role in the onset and progression of metabolic disorders. Our intention was to unravel their intricate dynamics and identify potential therapeutic targets. Thirdly, given the central role of dyslipidemia in MetS [4], we assessed circulating lipid profiles. Dyslipidemia’s contribution to MetS pathophysiology, including its role in promoting IR, atherosclerosis, and cardiovascular disease (CVD), also underscores the significance of this investigation. Fourthly, we explored the influence of PAC on the dysregulation of the gut–liver axis, examining bile acid composition irregularities. Both factors are implicated in the development of various metabolic disorders, such as MetS, metabolic dysfunction-associated steatotic liver disease (MASLD, previously non-alcoholic fatty liver disease), obesity, and type 2 diabetes [18,19]. Fifthly, we delved into intestinal microbiota and short-chain fatty acids (SCFAs) profiles, recognizing their intimate connection with metabolic health [20,21]. Specifically, we examined their interplay, which is intricately linked to glucose and lipid metabolism, insulin sensitivity and energy homeostasis. By addressing these multifaceted aspects, we aim to elucidate the broader spectrum of mechanisms through which PAC may exert their beneficial effects on metabolic health.

## 2. Materials and Methods

### 2.1. Purification and Characterization of PAC Polymeric Fraction

Cranberry powdered extract was obtained from Diana Food Canada (Champlain, QC, Canada). The methods for extracting PAC and a detailed description of the purified PAC polymeric fraction were reported in our previous study [14].

### 2.2. Animals

Eight-week-old C57BL/6J male mice (Charles River; Montreal, QC, Canada) were housed in a controlled environment (22 ± 1 °C; 12/12 h light–dark) with free access to food and drinking water. Following one week of acclimation on a standard diet, mice were fed either a Chow- (Teklad, 2018, Envigo, Indianapolis, IN, USA) or a high-fat, high fructose (HFHF)-regimen, consisting of a combination of a high-fat, high-sucrose diet (D17032403, Research Diets, New Brunswick, NJ, USA) as well as 30% fructose in drinking water, with daily PAC doses of 200 mg/kg body weight by gavage (HFHF + PAC). The Chow- and HFHF-nourished mice received the vehicle (water) by gavage in order to mimic the conditions in the HFHF + PAC group. Body weight gain and food intake were assessed twice a week. Stool samples were collected at weeks 0, 6, 9, and 11 and then immediately stored at −80 °C for further analysis (Supplementary Figure S1). After 12 weeks on dietary regimens, animals were fasted for 6 h, then anesthetized with isoflurane (1.5%), and euthanized by cardiac puncture exsanguination. Blood was drawn in EDTA-treated tubes and immediately centrifuged (3000× *g* 20 min at 4 °C) to separate plasma from cells. Intestinal segments and the whole liver were carefully collected and weighted. Each adipose tissue region (from perirenal, epididymal, inguinal, and mesenteric) was separately collected and weighed, allowing the distribution of the overall adipose tissue to be established. Subsequently, all the weights of the individual fat tissues were added together to determine the total amount of adipose tissue. All tissue samples were then immediately flash-frozen in liquid nitrogen and finally stored at −80 °C until further analysis. Samples from all tissues for *n* = 8 mice/group were collected and placed in TRIzol and then stored at −20 °C pending mRNA quantification or fixed in 10% neutral buffered formalin pending histological examination. The animal study protocol was approved by the Institutional Review Board of Research Centre/CHU Sainte-Justine (protocol code 756; date of approval: 7 January 2019).

### 2.3. Glucose Homeostasis and Lipid Profile

Blood was collected from the tails of mice at weeks 0, 6, and 10 following a 6-h fast. Glycemia was immediately measured with an Accu-Check glucometer. The remaining blood samples were placed in EDTA-treated tubes, and plasma was isolated, as described in the previous section. Plasma insulin, triglycerides (TG), and total cholesterol (TC) were assessed using commercial kits, as described previously [14]. The homeostatic model assessment for IR (HOMA-IR) index was then calculated using the following formula: fasting insulinemia (μUI/mL) × fasting glycemia (mM)/405.

### 2.4. Biochemical Analysis

Plasma samples (*n* = 8 mice/group) obtained at week 10 were used to measure high molecular weight (HMW) adiponectin concentrations with the Mouse HMW and Total Adiponectin ELISA kit (ALPCO, Salem, NH, USA). Plasma samples (*n* = 8 mice/group) obtained at sacrifice (week 12) were also assessed for inflammatory factors by a 32-multiplex cytokine array (Eve technologies, Calgary, AB, Canada). Plasma bile acids were assessed using liquid chromatography coupled with tandem mass spectrometry, as described previously [22].

### 2.5. Lipoprotein Quantification

#### 2.5.1. Ultracentrifugation

Fresh whole plasma samples (*n* = 8 mice/group) obtained at sacrifice (week 12) were pooled, then submitted to sequential gradient ultracentrifugation for lipoprotein separation using a TLA-110 rotor in an Optima^TM^ MAX Ultracentrifuge (Beckman Coulter Life Sciences Headquarters, Indianapolis, IN, USA). Briefly, centrifugations were operated at 4 °C for isolation of TG-rich lipoprotein (1.006 g/mL, 350,000× *g* 3 h), low-density lipoprotein (LDL; 1.063 g/mL, 350,000× *g* 3 h) and high-density lipoprotein (HDL; 1.21 g/mL, 70,000× *g* 48 h).

#### 2.5.2. FPLC Lipoprotein Separation

TC and esterified cholesterol (EC) were measured in plasma lipoprotein fractions that were separated by size exclusion chromatography using a Superose 6 column on a Fast Performance Liquid Chromatography (FPLC) system with a Model 500 pump from Waters (Milford, MA, USA) [23]. Following separation, lipoprotein fractions were frozen at −80 °C pending further analysis.

### 2.6. Tissue Lipid and Free Fatty Acids Analyses

Liver and jejunum specimens were homogenized, and lipids were isolated following Folch chloroform–methanol extraction and then submitted to analysis with commercial kits for TG and TC assessment [24]. FA composition of lipids was determined in samples obtained from solid organs (liver, jejunum), plasma, and mesenteric adipose tissue. Briefly, for solid tissue sample preparation, 0.1 g of liver or jejunum sample was homogenized in 1 mL of PBS-EDTA 4 mM. For plasma sample preparation, 50 μL of plasma was diluted with 50 μL of saline. For mesenteric adipose tissue preparation, 0.02 g of sample was homogenized, and lipids were then extracted overnight at 4 °C using a Folch chloroform–methanol extraction. After evaporation of the lower phase, lipids were resuspended in 600 μL of H_2_O. All samples were then subjected to transesterification and injected into a gas chromatograph (Agilent 7890 GC, Mississauga, ON, Canada) using a 90 m × 0.32 mm WCOT-fused silica capillary column VF-23ms coated with 0.25 μm film thickness (Agilent, Mississauga, ON, Canada), as previously described [25].

### 2.7. Histological Analysis

At the time of sacrifice, liver, colon, and mesenteric adipose tissue samples (*n* = 4 mice/group) were fixed in 10% neutral buffered formalin, dehydrated in gradient ethanol washing series, and embedded in paraffin. Tissue sections (3 μm thick) were obtained with a microtome and immediately stained with either Masson’s Trichrome, Hematoxylin/Phloxin/Safran (HPS) and examined under an optic microscope by a pathologist who was blinded to the experimental protocol. Images of stained tissues were captured by Zeiss Imager A1, and measurements were evaluated with the Axiovision software version 1.53a (https://www.micro-shop.zeiss.com/en/us/system/software+axiovision-axiovision+program-axiovision+software/10221/#variants, accessed on 12 July 2022) [26]. Specifically for mesenteric adipose tissue, automated analysis of histological images was performed using Adiposoft software version 1.16 plug-in (https://imagej.net/plugins/adiposoft, accessed on 12 July 2022) using the method developed by Galarraga et al. [27,28].

### 2.8. mRNA Isolation, Reverse Transcription, and Quantitative PCR Analysis

Intestinal and liver tissue samples were homogenized in TRIzol reagent, and total RNA was extracted. For each gene, mRNA expression was normalized to beta-actin as a reference gene, and expression levels were calculated using the 2^−∆∆CT^ method, as per our previous publications [14,24,28]. The primers used are listed in Supplementary Table S1.

### 2.9. 16S rRNA V6 Library Construction for Ion Torrent Sequencing and Analysis

16S rRNA amplicon libraries targeting the V6 hypervariable region were constructed and sequenced as previously described [28,29,30]. Briefly, the extracted metagenomic DNA was amplified in the PCR reaction using primers targeting the V6 region that contained an 11 BP barcode sequence and Ion Torrent sequencing adapter [28,31]. The demultiplexed and filtered reads for the HFHF and HFHF + PAC mice are now available in the NCBI Sequence Read Archive (SRA) under the project accession number PRJNA1156077. Raw reads for the Chow mice are available under accession PRJNA1016344. Amplicon sequence variants (ASVs) were identified using the DADA2 workflow, as previously described [32]. In brief, reads were quality-filtered to exclude those with more than one expected error. Pooled correction and chimera filtering were then performed following the recommended settings for Ion Torrent datasets. Taxonomy was assigned using the RDP Naive Bayesian Classifier algorithm, as implemented by DADA2 against the Silva 132 database. ASVs were inserted into the Silva 132 backbone tree using SEPP with the default settings [33].

### 2.10. Short Chain Fatty Acids Analysis

Total SCFA content was determined for mouse initial and final stools (weeks 0 and 11) in each feeding regime as previously described [28]. SCFA concentrations were calculated using a calibration curve using the internal standards. Samples with values below the quantitative limit of the assay and SCFAs with >90% of the samples below the detection limit were discarded from further analysis [28].

### 2.11. Statistical Analysis

#### 2.11.1. General Statistical Analysis

Data are expressed as means ± SEM. Following normal distribution assessment (Shapiro–Wilk test), statistical analysis was performed for the three group comparisons using one-way analysis of variance (ANOVA) with a post hoc Bonferroni multiple comparison test. All statistical analyses were performed on IBM SPSS version 29.0 (IBM, Armonk, NY, USA, 2023). Results were considered statistically significant at *p* < 0.05.

#### 2.11.2. Microbiome Statistical Analysis

Subsequent ASV analysis was conducted in R using phyloseq [34]. Contaminant ASVs were removed using the decontam R package, applying the frequency method and controlling for sequencing chips [35]. ASVs were subsequently filtered to keep those with ≥2 counts in at least 5% of the final sample dataset, and samples with <39,000 reads after filtering were removed from further analysis. Sample beta diversity was examined using the unweighted Unifrac distance, and significant clustering was assessed using PERMANOVA using “vegan” [36]. Differentially abundant ASVs over time between HFHF and HFHF + PAC were identified using metagenomeSeq’s fitTimeSeries function [37]. Multiple comparisons were controlled using the Benjamini and Hochberg approach, and the criterion for statistical significance was set at *p* < 0.05 with a differential abundance interval length > 1 week.

## 3. Results

### 3.1. PAC Hinders Diet-Derived Obesity and Is Reflected in Improved Adipocyte Histology

**Figure 1:**
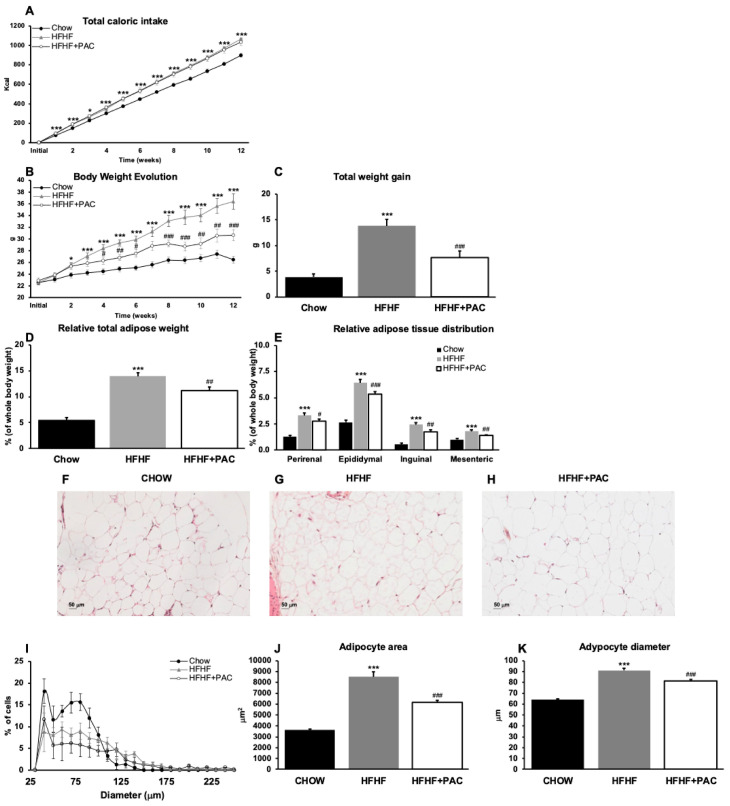
Effect of PAC on caloric intake, body weight gain, and adiposity. Mice were fed either a standard Chow diet or a high-fat, high-fructose diet (HFHF) ± 200 mg/kg polyphenol proanthocyanidin-rich fraction (PAC) body weight/day by gavage for 12 weeks. Chow diet and HFHF-fed mice were gavaged with a water vehicle. (**A**) energy intake, (**B**) body weight gain, and (**C**) total weight gain over the 12-week period. (**D**) Total adipose tissue weight (as a total of collected fat pads) and (**E**) its tissue distribution. Results are presented as means ± SEM for *n* = 12 mice/group. * *p* < 0.05, *** *p* < 0.001 vs. Chow; ^#^ *p* < 0.05, ^##^ *p* < 0.01, ^###^ *p* < 0.001 vs. HFHF mice. Representative images of adipocytes in mesenteric adipose tissue stained with HPS in mice fed (**F**) a Chow diet, (**G**) an HFHF diet, and (**H**) an HFHF + PAC diet are presented (magnification × 200, scale bar = 50 μm). (**I**) Adipocyte size distribution (%), (**J**) adipocyte area (μM^2^), and (**K**) adipocyte diameter (μM). Data are expressed as means ± SEM for *n* = 4 mice/group. *** *p* < 0.001 vs. Chow; ^###^ *p* < 0.001 vs. HFHF mice.

As noted in Figure 1A, caloric intake was inferior in the regular Chow-fed group as opposed to HFHF and HFHF + PAC groups. However, total energy intake did not differ between HFHF and HFHF + PAC groups. Nevertheless, PAC supplementation reduced HFHF-related obesity after 3 weeks of feeding and onward, a finding which gained significance after 4 weeks and remained significant after 6 weeks and onward (Figure 1B). After 12 weeks of treatment, PAC reduced HFHF-mediated weight gain (134%, *p* < 0.001) (Figure 1C). Similar findings were observed in total adiposity and fat mass distribution (Figure 1D,E). As mesenteric adipose tissue accumulation is most associated with MetS-related cardiometabolic disturbances and critically situated along the gut–liver axis, we aimed to examine HPS-stained histological sections of mesenteric adipose tissue in all three groups (Figure 1F–H). Histopathological analysis revealed that the HFHF diet led to an increase in the variability of adipocyte diameter, regardless of PAC administration, when compared to the Chow diet (Figure 1I). HFHF led to adipocyte hypertrophy, as noted by an increase in adipocyte surface area (235%; *p* < 0.001) and adipocyte diameter (142%; *p* < 0.001), findings that were reduced in the PAC-treated group (72% and 89%, respectively) (Figure 1J,K). Thus, our results show that in a MetS, HFHF-treated murine model, PAC exerts an anti-obesity effect, including protective ramifications to the adipose tissue profile.

### 3.2. PAC Prevents Glucose Dysmetabolism, Dyslipidemia, and Pro-Inflammatory Profile

**Figure 2:**
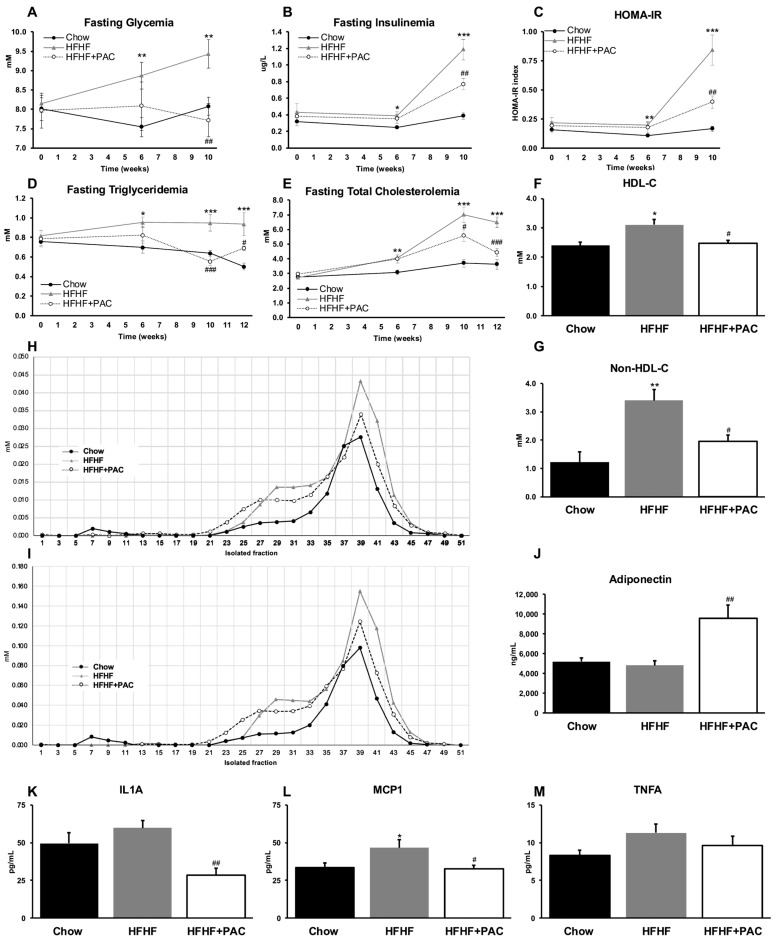
Effect of PAC on insulin resistance, fasting lipid profile, lipoprotein composition, and inflammatory circulating biomarkers. At weeks 0, 6, and 10, (**A**) fasting glycemia and (**B**) fasting insulinemia was measured, enabling the calculation of the (**C**) Homeostatic model assessment of insulin resistance (HOMA-IR). At weeks 0, 6, 10, and 12, (**D**) fasting triglyceridemia and (**E**) fasting total cholesterolemia were measured. Results are presented as means ± SEM for *n* = 6–12 mice/group. At week 12, the lipid profile was further investigated to determine levels of (**F**) HDL-cholesterol and (**G**) non-HDL-cholesterol. Results are presented as means ± SEM for *n* = 4 pooled plasma/group. At week 12, plasma from *n* = 8 mice/group was collected and pooled for lipoprotein determination. Isolated fractions were first obtained via fast-protein liquid chromatography (FLPC) and then were analyzed to reveal content in (**H**) total cholesterol and (**I**) esterified cholesterol, as described in Materials and Methods. Circulating (**J**) adiponectin was measured at week 10. Circulating (**K**) interleukin-1-alpha (IL1A), (**L**) Monocyte Chemoattractant Protein-1 (MCP1), and (**M**) Tumor Necrosis Factor-alpha (TNFA) were obtained at week 12. Results are presented as means ± SEM for *n* = 6–8 mice/group. * *p* < 0.05, ** *p* < 0.01, *** *p* < 0.001 vs. Chow; ^#^ *p* < 0.05, ^##^ *p* < 0.01, ^###^ *p* < 0.001 vs. HFHF mice.

We sought to explore the influence of PAC on fasting glycemia (Figure 2A) and insulinemia (Figure 2B) as well as HOMA-IR index calculation (Figure 2C) at weeks 0, 6, and 10. PAC-treated mice exhibited normoglycemia throughout the experiments and were significantly different from HFHF mice at week 10. Moreover, hyperinsulinemia was significantly, albeit modestly, reduced in PAC-treated mice, a finding that correlated more closely with HOMA-IR. Concomitantly, we explored PAC treatment on lipid profiles at weeks 0, 6, 10, and 12. Similar trends were seen with significant differences between HFHF and Chow groups as early as 6 weeks, and all three groups were found to be significantly different by 10 weeks and onward for TG (Figure 2D) and TC (Figure 2E) levels. At sacrifice (week 12), PACs were found to alleviate HFHF-mediated dyslipidemia with blunted levels of HDL-C (Figure 2F) and, more importantly, non-HDL-C (Figure 2G). These results are further illustrated by FPLC-led analysis of circulating lipoproteins content in TC (Figure 2H) and esterified cholesterol (Figure 2I). Prior to investigating further possible mechanisms, we looked at adipokine and pro-inflammatory cytokine markers in circulation.

Additional circulating inflammatory cytokines were also analyzed but did not show specific trends. When collectively combined into a cytoscore [38], the HFHF diet led to an increase in pro-inflammatory cytokines that correlated strongly with markers of Mets. However, this effect was dramatically reduced by PAC supplementation (Supplementary Figure S2).

Interestingly, PAC supplementation led to increased adiponectin concentrations in comparison to Chow and HFHF treatments (Figure 2J), while HFHF-mice exhibited the highest levels of circulating interleukin-1-alpha (IL1A) (Figure 2K), Monocyte chemoattractant protein-1 (MCP1) (Figure 2L) and Tumor Necrosis Factor-alpha (TNFA) (Figure 2M) when compared to Chow or HFHF + PAC mice.

### 3.3. PAC Relieve Hepatic Lipid Accumulation, Histological Markers of Inflammation, and Total Organ Weight

**Figure 3:**
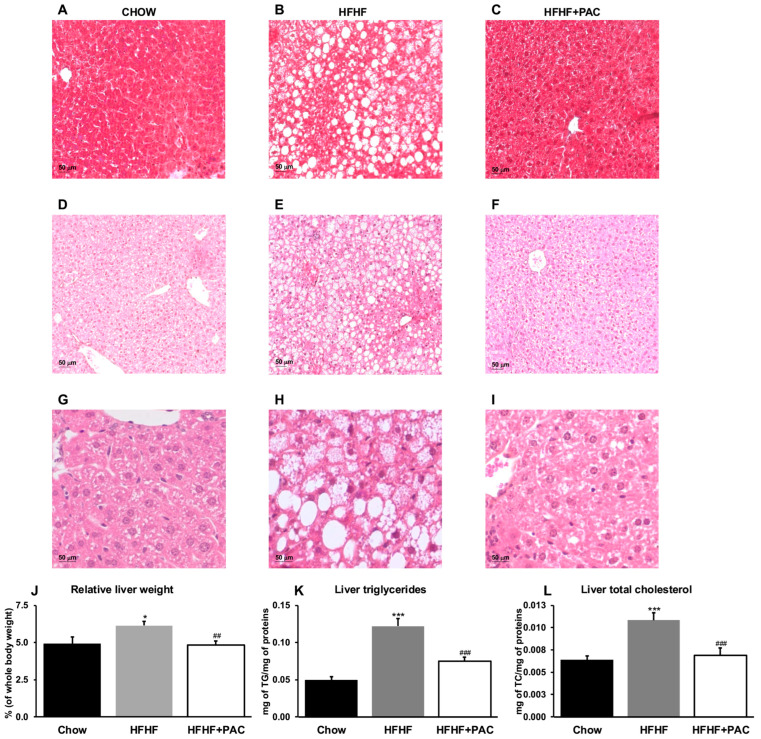
Effect of PAC on hepatic lipid accumulation. Fixed, paraffin-embedded liver sections from Chow, high-fat, high fructose (HFHF) and HFHF + PAC-fed mice stained either with (**A**–**C**) Masson’s trichrome (magnification × 200, scale bar = 50 μm) or HPS ((**D**–**F**) magnification × 50, scale bar = 50 μm; (**G**–**I**) magnification × 630, scale bar = 50 μm); representative images for *n* = 4 mice/group are presented. (**J**) Liver weights were measured, and (**K**) triglycerides and (**L**) total cholesterol contents were quantified in liver tissues for *n* = 6–8 mice/group. Results are presented as means ± SEM for *n* = 8 mice/group. * *p* < 0.05, *** *p* < 0.001 vs. Chow; ^##^ *p* < 0.01, ^###^ *p* < 0.001 vs. HFHF mice.

Hepatic histological examination of HFHF mice showed a vast number of fat droplets of different sizes, suggesting an increased lipid accumulation compared to the Chow group. However, PAC treatment reduced lipid accumulation, thereby alleviating hepatic steatosis (Figure 3A–F). Closer examination of histological markers indicative of hepatocellular ballooning, steatosis, fibrosis, and lobular inflammation revealed that PAC administration led to a non-diagnostic histological score of liver disease, while HFHF mice were aligned with a diagnosis of MASLD (Figure 3G–I and Supplementary Table S2). Lipid determinations confirmed these observations, given that PAC prevented the diet-related effects on total liver weight (Figure 3J), TG (Figure 3K), and TC (Figure 3L) hepatic content.

### 3.4. PAC Modulate Fatty Acid Composition Along the Gut–Liver Axis

**Figure 4:**
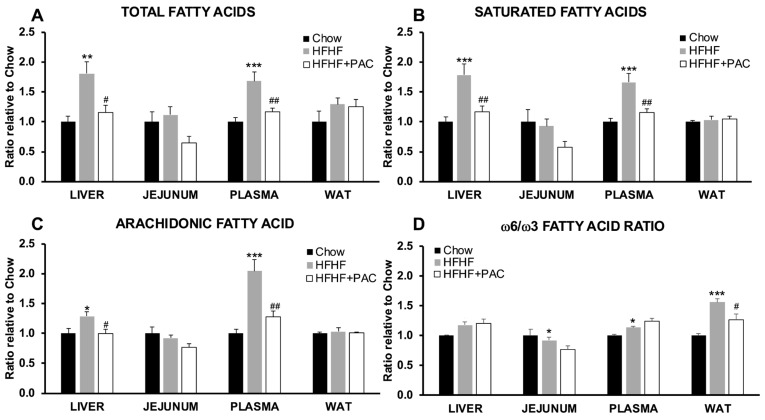
Effect of PAC of fatty acid composition across the gut–liver axis. Mice were fed either a standard Chow diet or a high-fat, high-fructose diet (HFHF) or HFHF + PAC for 12 weeks. Liver, jejunum, plasma, and mesenteric white adipose tissue (WAT) specimens were collected and submitted to high-performance liquid chromatography (HPLC) for fatty acid quantification of (**A**) total fatty acids, (**B**) saturated fatty acids, (**C**) arachidonic fatty acid and (**D**) ω-6/ω-3 calculated ratio. Data are expressed as means ± SEM relative to Chow for *n* = 6–8 mice/group. * *p* < 0.05, ** *p* < 0.01, *** *p* < 0.001 vs. Chow; ^#^ *p* < 0.05, ^##^ *p* < 0.01 vs. HFHF mice.

While similar observations were made for TG and TC content in jejunum tissue analysis (Supplementary Figure S3), we sought to further explore lipid metabolism along the gut–liver axis by scrutinizing FA composition among liver, jejunum, mesenteric white adipose tissue (WAT) as well as those circulating in plasma. Overall, HFHF led to an overall increase in both the total and saturated FAs in liver and plasma samples when compared to Chow mice, whereas PAC treatment prevented these trends (Figure 4A,B). No significant changes were observed in the gut or WAT. In order to identify pro-inflammatory FA markers, we also evaluated arachidonic (AA) FA level and omega-6/omega-3 (ω-6/ω-3) FA ratio (Figure 4C,D). The analysis revealed a similar trend to those observed for total and saturated FA, as the HFHF diet led to an increase in AA FA levels in both the liver and plasma, which was not observed in the jejunum and in WAT, while PAC reduced these effects. Results were more mitigated with the ω-6/ω-3 calculation. The ratio was increased in HFHF mice plasma and WAT but remained unchanged in the liver. Interestingly, PAC treatment resulted in a robust decrease in the WAT ω-6/ω-3 ratio when compared to HFHF mice.

### 3.5. PAC Effect on Gut Histology

**Figure 5:**
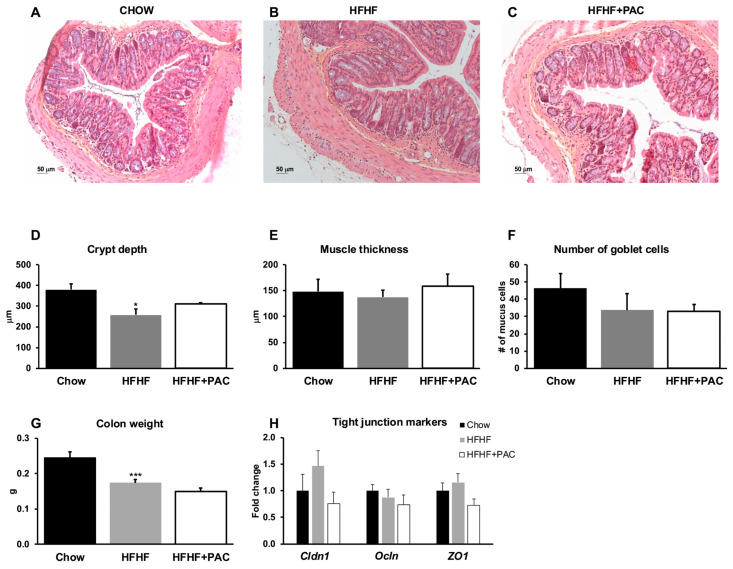
Effect of PAC on gut histology. Cross-sections of distal colon tissue were stained with HPS to evaluate the impact of PAC supplementation on intestinal morphology. Representative images of colon sections in mice fed (**A**) a Chow diet, (**B**) a high-fat, high-fructose (HFHF) diet, and (**C**) an HFHF + PAC diet are presented (magnification × 25, scale bar = 50 μm). Histological parameters assessed included (**D**) crypt depth, (**E**) muscle thickness, and (**F**) number of goblet cells per crypt; data are shown as the means ± SEM for *n* = 4 mice/group. (**G**) Colon weight was measured and expressed as the means ± SEM relative to Chow for *n* = 12 mice/group. mRNA was extracted from distal colon specimens, and gene expression of tight junction markers (**H**) claudin 1 (*Cldn1*), occludin (*Ocln*), and zonula occludens-1 (*ZO1*) were measured and expressed as the means ± SEM relative to Chow for *n* = 6–8 mice/group. * *p* < 0.05, *** *p* < 0.05 vs. Chow.

Distal colon sections were stained with HPS for histological assessment (Figure 5A–C). PAC supplementation did not significantly blunt the HFHF-related reduction in crypt depth (Figure 5D). No meaningful variation was otherwise noted with respect to muscle thickness (Figure 5E) or goblet cell prevalence (Figure 5F). The HFHF diet led to a macroscopic hypotrophy of total colon weight, a finding that was not impacted by PAC supplementation (Figure 5G). Lastly, measuring tight junction genes (claudin 1, occludin, and zonula occludens-1) did not yield any significant differences among the three animal groups (Figure 5H).

### 3.6. PAC Alleviate Inflammation, ER Stress, and Lipid Dysregulation Along the Gut–Liver Axis

**Figure 6:**
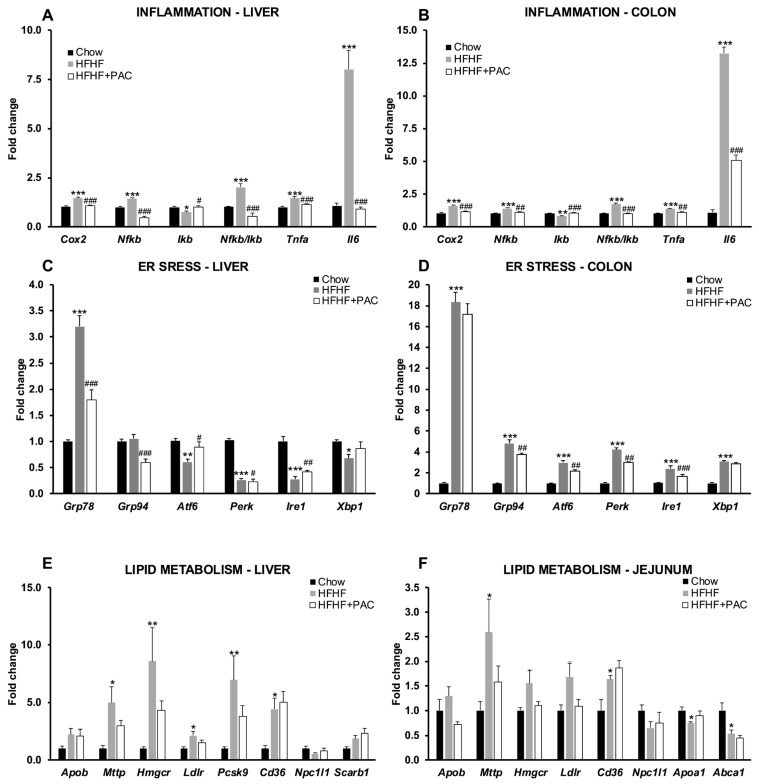
Effect of PAC on inflammatory markers, reticulum stress markers, and lipid metabolism in the gut–liver axis. mRNA was extracted from liver, distal colon, and jejunum specimens in Chow-, high-fat, high-fructose (HFHF) and HFHF + PAC-fed mice. (**A**) Liver and (**B**) distal colon inflammation was evaluated through gene expression of cyclooxygenase-2 (*Cox2*), nuclear factor kappa B (*Nfkb*), inhibitor of kappa B (*Ikb*), the *Nfkb*/*Ikb* ratio, Tumor Necrosis Factor-alpha (*Tnfa*) and interleukin-6 (*Il6*). (**C**) Liver and (**D**) distal colon endoplasmic reticulum stress was evaluated through gene expression of Glucose-regulated protein 78 kDa (*Grp78*), Glucose-regulated protein 94 kDa (*Grp94*), Activating transcription factor 6 (*Atf6*), Inositol-requiring protein-1 (*Ire1*), Protein kinase RNA-like ER kinase (*Perk*) and X-box-binding protein (*Xbp1*). Liver and jejunum lipid metabolism was evaluated through gene expression of (**E**,**F**) Apolipoprotein B (*Apob*), (**E**,**F**) Microsomal triglyceride transfer protein (*Mttp*), (**E**,**F**) 3-hydroxy-3-methylglutaryl-Coenzyme A reductase (*Hmgcr*), (**E**,**F**) LDL receptor (*Ldlr*), (**E**) Proprotein convertase subtilisin (*Pcsk9*), (**E**,**F**) cluster of differentiation antigen 36 (*Cd36*), (**E**,**F**) Niemann-Pick C1-like 1 (*Npc1l1*), (**E**) Scavenger receptor class B, member 1 (*Scarb1*), (**F**) Apolipoprotein A1 (*Apoa1*), and (**F**) ATP-binding cassette transporter 1 (*Abca1*). Data are expressed as the means ± SEM relative to Chow for *n* = 6–8 mice/group. * *p* < 0.05, ** *p* < 0.01, *** *p* < 0.001 vs. Chow; ^#^ *p* < 0.05, ^##^ *p* < 0.01, ^###^ *p* < 0.001 vs. HFHF mice.

Gene expression of inflammatory markers cyclooxygenase-2 (*Cox2*), nuclear factor kappa B (*Nfkb*), inhibitor of kappa B (*Ikb*), and the resulting *Nfkb*/*Ikb* ratio, *Tnfa* and interleukin-6 (*Il6*) were measured in both liver (Figure 6A) and colon (Figure 6B). Overall, all markers demonstrated a clear and significant increase in pro-inflammatory levels due to the HFHF diet. Strikingly, PAC supplementation prevented the increase in pro-inflammatory expression for all investigated genes. Considering its association with MetS, we next turned to endoplasmic reticulum (ER) stress key markers. Hepatic expression of *Grp78* and *Grp94* was blunted by PAC treatment, while trends toward increased ER stress were observed for *Atf6*, *Ire1*, *Perk*, and *Xbp1* (Figure 6C). Interestingly, the colon of HFHF mice exhibited increased overall ER stress-related mRNA expression of glucose-regulated protein 78 kDa (*Grp78*), 94 kDa (*Grp94*), activating transcription factor 6 (*Atf6*), inositol-requiring protein-1 (*Ire1*), protein kinase RNA-like ER kinase (*Perk*) and X-box-binding protein-1 (*Xbp1*) markers compared to the liver, and further highlighted the local protective effects conferred by PAC (Figure 6D). Lastly, we sought to investigate key lipid metabolism regulators along the gut–liver axis. In the liver (Figure 6E), these included markers for lipoprotein assembly [apolipoprotein B (*Apob*), microsomal triglyceride transfer protein (*Mttp*)], de novo cholesterol synthesis (*Hmgcr*) and cholesterol flux homeostasis [(LDL receptor (*Ldlr*), proprotein convertase subtilisin/kexin type 9 (*Pcsk9*), cluster of differentiation antigen 36 (*Cd36*), Niemann-Pick C1-like 1 (*Npc1l1*) and scavenger receptor class B, member 1 (*Scarb1*)]. HFHF diet led to a significant increase in liver gene expression of *Mttp*, *Hmgcr*, *Ldlr*, *Pcsk9*, and *Cd36*, and more modest trends in the remaining markers. Despite downward trends in these markers, PAC supplementation did not reveal any significant differences. Since the jejunum is the primary site for intestinal lipid absorption, we explored similar markers while adding *Apoa1* and ATP-binding cassette transporter 1 (*Abca1*) to analyze HDL metabolism (Figure 6F). Overall, only modest trends were observed, except for increased gene expression of *Mttp* and *Cd36*, in addition to a significant decrease in *Apoa1* mRNA levels. While PAC did not reveal significant differences from the HFHF animal group, gene expression for *Apob*, *Mttp*, *Ldlr*, and *Apoa1* remained unchanged.

### 3.7. PAC Influences Bile Acid Metabolism in the Gut–Liver Axis

**Figure 7:**
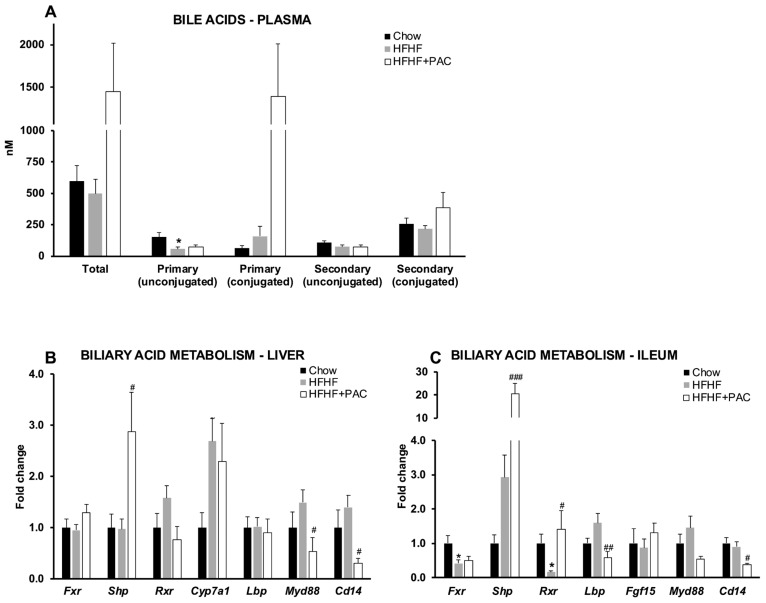
Effect of PAC on bile acid metabolism. (**A**) Bile acid pool size and composition were assessed in mice plasma, as described in the Materials and Methods section. Messenger RNA was extracted from liver and ileum specimens in Chow-, high-fat, high-fructose (HFHF), and HFHF + PAC-fed mice. Liver and ileum bile acid metabolism was evaluated through gene expression of (**B**,**C**) Retinoid X receptor (*Rxr*), (**B**,**C**) Small heterodimer partner (*Shp*), (**B**,**C**) Myeloid differentiation primary response 88 (*Myd88*), (**B**,**C**) Lipopolysaccharide-binding protein (*Lbp*), (**B**,**C**) Farnesoid X receptor (*Fxr*), (**B**) Cholesterol-7-alpha-hydroxylase (*Cyp7a1*), (**C**) Fibroblast growth factor 15 (*Fgf15*) and (**B**,**C**) *Cd14*. Data are expressed as the means ± SEM relative to Chow for *n* = 6–8 mice/group. * *p* < 0.05 vs. Chow; ^#^ *p* < 0.05, ^##^ *p* < 0.01, ^###^ *p* < 0.001 vs. HFHF mice.

Bile acids regulate their own transport and metabolism through the entero-hepatic circuit but also represent a promising avenue for regulating glycemic and lipid metabolism. PAC-supplemented mice displayed an increased, albeit non-statistically significant, trend in circulating total bile acids, while Chow and HFHF-fed mice displayed similar levels. Accordingly, we observed some changes in specific bile acids but without significant differences between groups (Figure 7A). We then examined the expression of retinoid X receptor (*Rxr*), small heterodimer partner (*Shp*), myeloid differentiation primary response 88 (*Myd88*), lipopolysaccharide-binding protein (*Lbp*), farnesoid X receptor (*Fxr*) and *Cd14* in both the liver (Figure 7B) and ileum (Figure 7C). Cholesterol-7-alpha-hydroxylase (*Cyp7a1*) and fibroblast growth factor 15 (*Fgf15*) were only examined in the liver and ileum, respectively, due to their tissue-specific expression. PAC promoted a significant upregulation of *Shp* while blunting *Cd14* in the liver and ileum. While hepatic *Rxr* gene expression showed a downward trend following PAC supplementation, ileal *Rxr* expression was significantly upregulated. Contrary to the hepatic expression of *Lbp*, we further observed an inhibition of ileal *Lbp* mediated by PAC treatment. Finally, while HFHF led to significant blunting of ileal *Fxr*, PAC supplementation did not correlate with any modification.

### 3.8. PAC Alter Microbiota Composition and Is Reflected in SCFAs Levels

**Figure 8:**
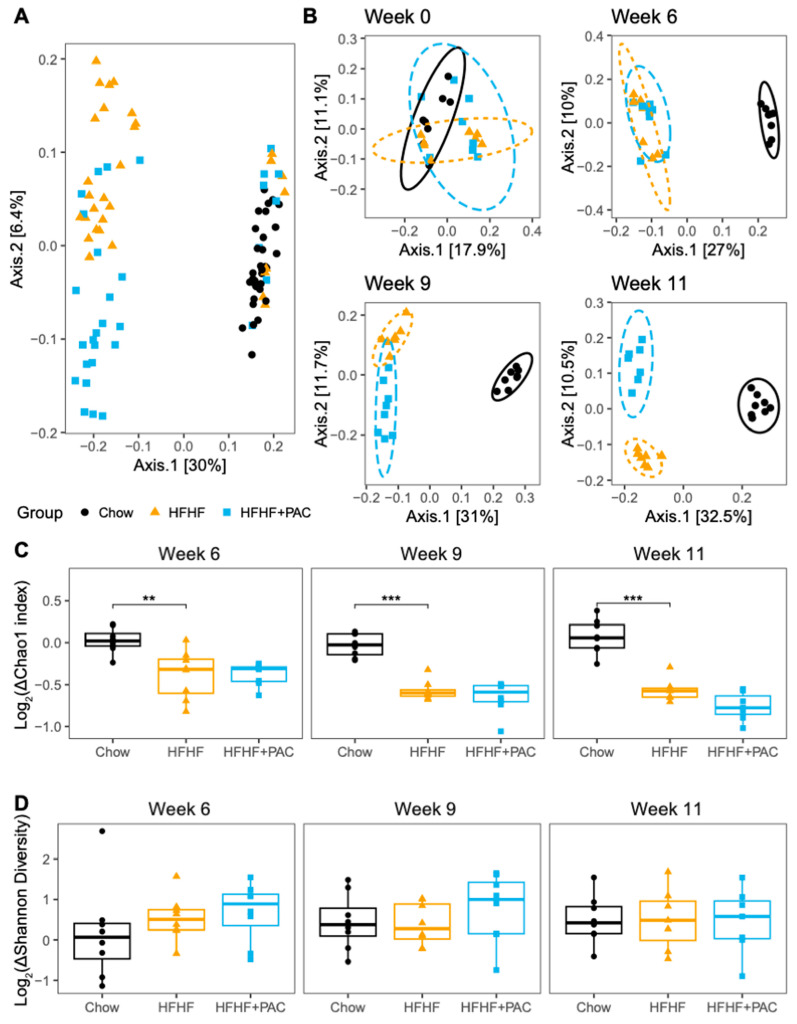
HFHF + PAC diet causes clear shifts to mouse microbiomes over time. Principal coordinate analysis using the unweighted Unifrac distance on all mouse microbiota (**A**) and each time point analyzed separately (**B**). Initial mouse microbiota all cluster together (Week 0; (**A**,**B**)) with HFHF diet, causing dramatic shifts in microbiota composition over time, while Chow-fed mice remain relatively consistent. Changes in species richness (Chao 1 index; (**C**)) and evenness (Shannon diversity; (**D**)) over time for each mouse group normalized to T0. HFHF mice have reduced microbial richness as compared to Chow. There was no significant change in species evenness throughout the experiment. Ellipses in B represent the 95% confidence intervals for each group. *n* = 8 mice/group. ** *p* < 0.01, *** *p* < 0.001 vs. Chow.

While Chow, HFHF, and HFHF + PAC mouse microbiota were initially indistinguishable at the beginning of the intervention, the HFHF and HFHF + PAC mice showed an immediate shift from Chow-fed mice once the diet regimes began (Figure 8A,B). Moreover, the HFHF and HFHF + PAC microbiota became progressively more distinct during the intervention, reaching complete separation by week 11. This indicates that PAC treatment had a progressive impact on microbiota composition that builds over time. Both HFHF and HFHF + PAC mice microbiota showed a decrease in the number of species present as compared to baseline (Figure 8C).

**Figure 9:**
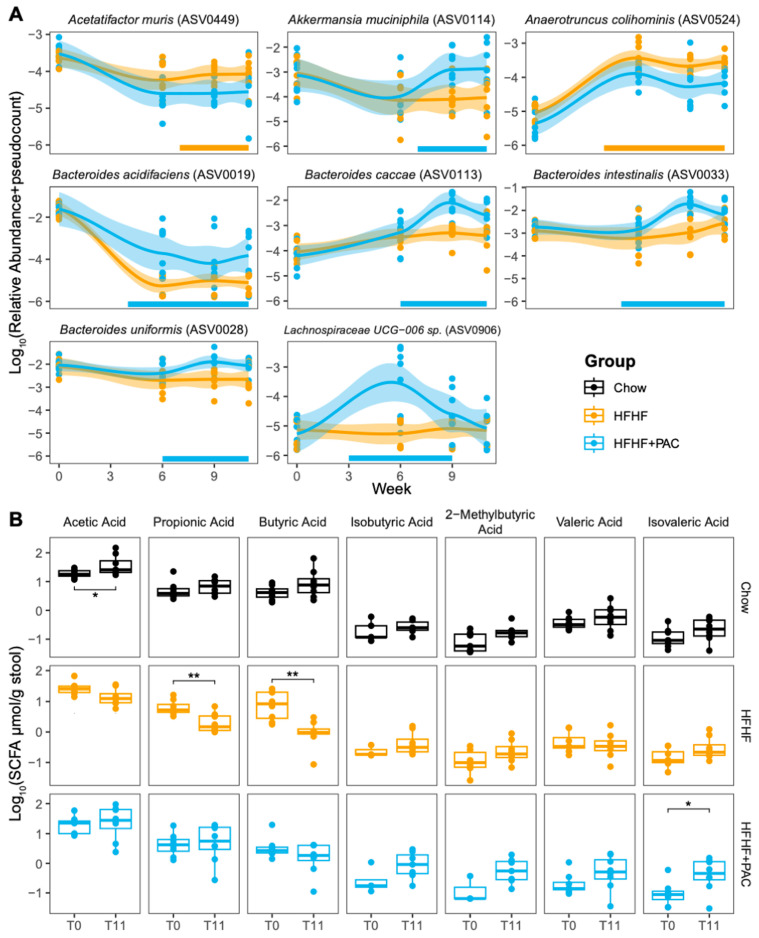
PAC treatment results in the enrichment of specific bacterial species and increases in specific SCFAs when compared to baseline. (**A**) Selected amplicon sequence variants (ASVs) annotated and defined at the species level with differentially abundant changes over time. ASV relative abundance was log_10_ transformed with a pseudocount added as required. Points represent each sample, with lines representing LOWESS fits and shaded regions denoting 95% confidence intervals. Bars along the *x*-axis highlight the time spans identified as showing different abundance patterns. The colour represents which group was enriched for each time period. (**B**) Levels of measured SCFAs in each feeding group at T0 and after 11 weeks of the dietary intervention (see Supplementary Table S3 for overall variation between both endpoints (TΔ) in total SCFA and individual components). Boxplots show the interquartile range with the median represented as a line. *n* = 8 mice/group. * *p* < 0.05, ** *p* < 0.01 vs. different timepoints.

The analyses were also able to identify several specific microbial species with evolving abundance over time and differentially between the HFHF and HFHF + PAC mice. In particular, we observe an abundance in *Akkermansia muciniphila* and several *Bacteroides* genera (*B. acidifaciens*, *B. caccae*, *B. intestinalis* and *B. uniformis*) in the HFHF + PAC mice (Figure 9A).

Collectively, these changes in species composition were reflected by an increasing ratio of Firmicutes/Bacteroides in HFHF-fed mice over time, which PAC supplementation prevented (Supplementary Figure S4). To further investigate microbial function, we investigated fecal SCFA composition at onset and experiment completion (Supplementary Table S3, Figure S5). PAC supplementation prevented the reduction in SCFA production observed in HFHF-fed mice. For individual SCFA, there was only a significant increase in isovaleric acid levels in HFHF + PAC mice as compared to the baseline, with a trend for rises in valeric acid (Figure 9B).

## 4. Discussion

Polyphenols are increasingly recognized as a promising tool for treating and preventing complex diseases, offering enhanced nutritional support and targeted health benefits. These natural compounds are known to lower disease risk factors, boost immune health, and aid in managing chronic conditions such as CVD, diabetes, and cancer [39,40,41].

Polyphenols align seamlessly with personalized nutritional habits and have the potential to complement traditional medical treatments, contributing to a more comprehensive approach to health care. Continuous research is revealing new beneficial properties of polyphenols, increasing their potential. As discussed in our previous work [14], the daily dose of 200 mg/kg in mice translates to approximately 16 mg/kg/day when converted for humans. Considering an average individual weighing 70 kg, this corresponds to an intake of 1120 mg/day of PAC, or roughly 350 g of cranberries. While this may appear as an excessive dose, it is crucial to stress that the aim of this paper was to investigate a purified extract of PAC polymers. Observational studies in humans with the aim of estimating polyphenol intake in diet are marked by wide variability [42,43,44]. Indeed, a recent multicenter study across Europe and the Americas performed by Noronha et al. suggested a total polyphenol intake of 300–1700 mg per day [44]. Therefore, while the extract used in this study was exclusively made of purified PAC polymers, the overall dose used is comparable to an average polyphenol intake.

While polyphenols have shown promising health outcomes, there are various limitations and aspects to bear in mind. The available information is primarily derived from experiments involving fruits and plant extracts, which contain multiple classes of polyphenols and other additional substances, based on tests conducted on various cellular models. However, the present preclinical study focuses on purified PAC, characterized by a high degree of polymerization, with significant bioactivity despite absent bioavailability. As defined in our previous work [14], the PAC used in this study stems from a highly purified fraction with a high degree of polymerization rather than a whole cranberry extract containing various polyphenol fractions. Using the purified fraction, a recent study performing in vitro fecal batch fermentation on 34 human donors has shown that PAC was impervious to gut microbial degradation [45]. Additionally, polymeric A-type PACs with a degree of polymerization above 8 are poorly metabolized by the gut microbiota into bioavailable metabolites. Indeed, findings suggested that less than 1% of the ingested polymers can be metabolized by the microbiota, and only the monomers and dimers of flavan-3-ols can metabolized to some extent to produce characteristic metabolites like 3-4-dyhydroxyphenyl-valerolactone [45,46]. Importantly, our work differs from previous studies in that we utilized highly purified PAC rather than whole fruit extract.

Using a MetS mouse model, our recent findings have already demonstrated the potent ability of this highly polymerized PAC fraction to protect against obesity, IR, and hyperlipidemia [14]. Additionally, our data highlighted the capacity of PAC to mitigate OxS and inflammation. The objective of this work was to further elucidate the mechanisms of action of PAC along the gut–liver axis in MetS conditions. We conducted a comprehensive examination of (i) regional adipose tissue composition and adipocyte size in relation to obesity, glucose metabolism, and hyperinsulinemia; (ii) inflammation and ER stress linked to IR, hypertriglyceridemia, and hypercholesterolemia; and (iii) pro-atherosclerotic status associated with hyperlipidemia. Furthermore, we investigated the gut microbiota, SCFAs, and bile acids in relation to the gut–liver axis, which plays a crucial role in maintaining metabolic integrity.

The first experimental steps in this work uncovered the clear ability of PAC to significantly avert obesity by curbing weight gain during continual intake of a calorie-dense diet. This effect arises from a decrease in adipose tissue hypertrophy characterized by a reduction in adipocyte diameter, thereby limiting the accumulation of excess lipids.

In vitro, PAC suppresses adipogenesis in adipocytes by inhibiting transcription factors like PPARγ (Peroxisome Proliferator-Activated Receptor Gamma) and C/EBPα (CCAAT/Enhancer-Binding Protein-Alpha) [47]. As driving factors for adipogenesis, their suppression by PAC has been shown to limit preadipocyte differentiation into mature adipocytes [48,49,50]. Evidence is also available to stress that polyphenols activate the Wnt pathway, which inhibits adipogenesis by suppressing PPARγ and C/EBPα [51]. Ultimately, these modifications may play a driving part in modulating adipokine production, with systemic repercussions on metabolism thereafter [52,53]. PACs are also able to reduce lipid accumulation by stimulating lipolysis, inhibiting lipogenic enzymes, and activating AMP-activated protein kinase (AMPK) to enhance lipid catabolism [54,55,56,57]. In fact, polyphenols have been associated with the enhanced breakdown of TG into free FA, reducing intracellular lipid accumulation in adipocytes [47]. On the other hand, by downregulating lipogenic enzymes (e.g., fatty acid synthase and acetyl-CoA carboxylase), PAC has the capacity to reduce the synthesis of FA and TG [58,59]. Finally, PAC may activate AMPK, which inhibits lipogenesis and promotes lipid catabolism [60,61,62]. PAC may decrease the secretion of pro-inflammatory cytokines and enhance anti-inflammatory markers [63,64], thereby improving adipose tissue function and reducing ectopic lipid deposition. In addition, the potent antioxidant properties of PAC [65,66] protect adipocytes from OxS, which is a common feature of dysfunctional adipose tissue in obesity and MetS [67]. By reducing OxS, PAC helps maintain proper adipocyte function and insulin sensitivity [68,69]. Therefore, polyphenols can modulate adipocyte differentiation and lipid storage by targeting key signaling pathways and transcription factors involved in adipogenesis, lipolysis, and lipogenesis. These effects position PAC as promising compounds for managing obesity and improving metabolic health. To our knowledge, this is the first demonstration of PAC preventing visceral adipocyte hypertrophy in the setting of MetS. By restricting the enlargement of fat cells and preventing the accumulation of adipose tissue, especially in visceral adipose tissue, PAC thus appears as an important factor in mitigating the development of related health issues, including obesity, MetS, MASLD, diabetes, and CVD [70,71,72].

As glucose dysmetabolism is central to MetS development and progression, validation that this process could be modulated was critical to substantiate PAC’s advantageous effects. In fact, PAC administration successfully countered the rise in blood glucose and insulin levels mediated by HFHF. Preserved insulin sensitivity was observed following HOMA-IR calculation, demonstrating the moderating influence of PAC on IR, which is a hallmark of MetS and related complications [73,74].

Considering the ability of PAC to ameliorate IR, we subsequently focused on exploring their mechanisms of action. In particular, we were interested in whether PAC impacted inflammation, as this has been shown to cause IR via the release of key pro-inflammatory markers, which impair the function of the insulin receptor on cell membranes, thereby reducing the power of insulin to bind effectively and activate its signaling pathways [74]. Our results show that PAC prevents the release of pro-inflammatory cytokines into the bloodstream as compared to HFHF conditions. As adipose tissue releases various signaling molecules known as adipokines, we assessed adiponectin levels, given its well-documented anti-inflammatory properties and role in IR [75,76]. In contrast to the blunted levels of pro-inflammatory IL1A, MCP1, and TNFA, adiponectin levels were increased, which strengthens the hypothesis that PAC has anti-inflammatory properties and insulin-sensitizing actions, potentially through its gut-derived mediation. It is worth noting that one of the mechanisms by which PAC could diminish IR may be through their control of adipose tissue mass and the downstream prevention of the pro-inflammatory phenotype derived from increased adipose tissue (especially visceral fat) stores. Thus, associated metabolic dysfunctions leading to IR may be avoided by normalizing inflammatory levels [77,78].

The consumption of HFHF has been found to modulate endogenous lipid profiles, as noted by the rise in hypertriglyceridemia and hypercholesterolemia. The treatment with PAC significantly reduced the plasma levels of TG and TC, particularly those of non-HDL-C, an important marker of CVD, especially in subjects with MetS [79,80]. PAC-induced hypolipidemic activity is probably due to decreasing lipoproteins. PACs may have a dual action: they can limit the release of FA from adipose tissue by decreasing its fat mass and preserving its regulation by insulin while also impacting the liver to reduce lipid overload as well as very low-density lipoprotein synthesis and secretion. To further deepen our understanding of PAC-potential properties on lipid metabolism and in view of the crucial role of the FA profiles in the development of MetS [81], we analyzed FA composition throughout the gut–liver axis in the plasma, liver, intestine, and visceral adipose tissue. PAC showed high potency to lower the absolute content of total FA, saturated FA, and AA FA in all the compartments except adipose tissue. PAC supplementation also revealed altered FA ratios in visceral fat. These decreases in total and saturated FA are indicative of reduced risk for IR, MetS, and MASLD, as well as CVD [82]. On the other hand, a lower AA FA level may mean better control of persistent low-grade inflammation, a hallmark of MetS, given the rapid conversion of AA FA into various inflammatory eicosanoids by the cyclooxygenase and lipoxygenase pathways [83]. Furthermore, disproportionally high levels of ω-6 FA compared to ω-3 FA can further contribute to eicosanoid pathways and exacerbate pro-inflammatory phenotypes [84].

As referenced before, the appraisal of plasma inflammatory markers was necessary for establishing systemic inflammatory conditions and providing a broad overview of the body’s inflammation status. Nevertheless, it was also important to determine the local markers in specific organs, such as the liver and intestine, to provide insights into the extent and nature of inflammation within these tissues. Another layer to this issue is the critical role of inflammatory factors in the connection between the gut and liver within the gut–liver axis under dysmetabolic conditions [85]. Gut-derived factors such as lipopolysaccharides and cytokines can induce liver inflammation, while liver responses can further affect gut health, creating a complex interplay that influences systemic inflammation and disease development [77]. This is where the remarkable anti-inflammatory potential of PAC becomes evident, as they significantly reduce *Cox2*, *Tnfa*, *Il6*, and *Nfkb* (contrary to *Ikb*), thereby resulting in a reduced *Nfkb*/*Ikb* ratio in both the liver and the colon. Similarly, PAC demonstrated their ability to act on components of ER stress (*Gpr78/94*, *Atf6*, *Ire1*, *Xbp1*), which is closely associated with inflammation. Therefore, targeting inflammation in both the gut and liver with PAC may be effective in managing metabolic disorders associated with the gut–liver axis. This holds particular importance since PAC may advantageously influence lipid metabolism, which is intricately linked to the gut–liver axis through processes involving absorption (e.g., *Apob* and *Mttp*), transport (e.g., *Lldr* and *Pcsk9*) and metabolism (e.g., *Hmgcr*). It is worth noting that the condition of the animals’ livers improved substantially with the administration of PAC.

Next, we sought to further investigate possible upstream factors that could influence the gut–liver axis in response to PAC supplementation. As our previous study had demonstrated that PAC could blunt metabolic endotoxemia in the context of diet-altered dysbiosis [14], we expanded our investigations to bile acid metabolism, SCFA, and gut microbiota composition. PAC supplementation resulted in a trend for increased circulating levels of total bile acids and of several specific subtypes. However, this observation was not statistically significant. Regarding downstream bile acid modulation, PAC led to a markedly upregulated expression of *Shp* in both the liver and ileum despite the absence of *Fxr* modulation. In keeping with other studies that observed a similar lack of *Fxr* modulation by PAC, including its downstream targets [86], potential inhibition of de novo lipogenesis may occur through *Rxr* activation or liver X receptor impediment [87]. This is unexpected, as upregulation of *Shp* would be expected to reduce the intake of bile salts due to the inhibition of ileal apical sodium-dependent bile salt transporter expression [88]. However, these findings suggest that PAC may promote *Shp* through an alternative pathway or potentially through a downstream metabolite.

In contrast with the PAC-led reversal of host-associated symptoms of MetS, PAC supplementation was unable to reverse the microbial alterations in terms of species richness induced by HFHF feeding, with both HFHF and HFHF + PAC-fed mice differing substantially from their Chow-fed counterparts. Remarkably, however, PAC provided unique signatures in species composition. Indeed, *A. muciniphila* showed an increase over time in response to PAC supplementation, and this microbe has been shown to be stimulated by the consumption of dietary phenols and repeatedly associated with beneficial health impacts [89,90]. In addition, numerous *Bacteroides* species showed increasing relative abundances over time during PAC supplementation, and these species are known to be involved in modifying bile acids into various bioactive forms, many of which have only recently been discovered to exist [91,92,93]. Moreover, the SCFA profile showed a significant decrease in propionic/butyric acid levels in HFHF mice, suggesting that PAC has prevented the depletion of microbes involved in these processes. Since the HFHF diet lacked any source of complex carbohydrates, it is reasonable to suggest that PAC could act as a fuel for SCFA production by the gut microbiota. Accordingly, this correlated with the SCFA production seen with PAC supplementation; these results notably contrast with investigations using other polyphenolic extracts, particularly those with increased bioavailability [94]. Nonetheless, SCFA production by the microbiota offers a mechanistic lever on which PAC could act, locally attenuating inflammation and thus promoting the preservation of intestinal homeostasis [95]. Moreover, at the systemic level, SCFAs such as acetate could impact the modulation of lipid metabolism and hepatic insulin sensitivity through an AMP-activated protein kinase α-dependent mechanism [96,97,98]. Additionally, the production of adiponectin in adipose tissue could also be influenced by SCFAs through GPR41/43 receptors [99,100]. Lastly, decreases in butyrate production and/or associated bacterial producers have been frequently observed in the context of MetS and other related diseases, such as obesity. Moreover, the observed decreases are often reversed upon therapeutic interventions [101,102]. For example, the anti-diabetic drug metformin has well-established impacts on gut microbiota composition and has been reported to increase the levels of butyrate producers in treated individuals [103].

## 5. Conclusions

In conclusion, PAC supplementation in an HFHF diet significantly reduced adipose tissue accumulation, attenuated hyperglycemia/hyperinsulinemia, exerted a protective effect against liver steatosis, prevented dyslipidemia, and promoted favourable lipoprotein composition. PAC also mitigated key risk factors associated with CVD and exhibited robust anti-inflammatory properties. PAC also impacted gut microbiota composition and functional outputs, with additional potential effects on bile acid levels/composition. Further studies are required to further elucidate these cascades, as these would deepen our understanding of how PAC prevents dysregulation in lipid metabolism and mitigates organ steatosis in the gut–liver axis, ultimately inhibiting MetS. Indeed, it is likely that the pleiotropic effects of PAC target the host, the microbiota dysbiosis, and the host-microbiota interactions via multiple distinct mechanisms. These highly polymeric compounds provide key insights into microbiota and gut-derived mechanisms in the prevention of MetS. Understanding these mechanisms will enable targeted therapeutic strategies for addressing these gut- and liver-derived metabolic disorders.

## Data Availability

All data supporting the findings of this study are available within the paper and its Supplementary Information. Raw data can be shared upon request by contacting Francis Feldman at francis.feldman@umontreal.ca.

## Supplementary Materials

The following supporting information can be downloaded at https://www.mdpi.com/article/10.3390/antiox14030268/s1. Figure S1: Experimental design; Figure S2: Effect of PAC on inflammatory circulating biomarkers and relationship with anthropometric and metabolic parameters; Figure S3: Effect of PAC on gut lipid accumulation; Figure S4: Firmicutes/Bacteroides ratio over time; Figure S5: Levels of measured SCFAs for each group at T0 or after 11 weeks of the dietary intervention; Table S1: List of primers used for RT-qPCR analysis; Table S2: Histology score of liver disease; Table S3: Concentrations of fecal SCFA.

## Abbreviations

AA. Arachidonic acid; *Abca 1*. ATP-binding cassette transporter 1; AMPK. AMP-activated protein kinase; ASVs. Amplicon sequence variants; Apo. Apolipoprotein; *Atf6*. Activating transcription factor 6; *Cldn1*. Claudin 1; *Cd36*. Cluster of differentiation antigen 36; *Cox2*. Cyclooxygenase-2; CVD. Cardiovascular disease; *Cyp7a1*. Cholesterol-7-alpha-hydroxylase; ER. Endoplasmic reticulum; FA. Fatty acid; *Fgf15*. Fibroblast growth factor 1; *Fxr*. Farnesoid X receptor; *Grp78*. Glucose-regulated protein 78 kDa; *Grp94*. Glucose-regulated protein 94 kDa; HDL. High-density lipoprotein; HFHF. High-fat/high-fructose; *Hmgcr*. 3-hydroxy-3-methylglutaryl-Coenzyme A reductase; HMW. High molecular weight; HOMA-IR. Homeostatic model assessment for IR; HPS. Hematoxylin/Phloxin/Safran; *Ikb*. Inhibitor of kappa B; Il. Interleukin; IR. Insulin resistance; *Ire1*. Inositol-requiring protein-1; *Lbp*. Lipopolysaccharide-binding protein; LDL. Low-density lipoprotein; *Ldlr*. LDL receptor; MASLD. Metabolic dysfunction-associated steatotic liver disease; MCP1. Monocyte chemoattractant protein-1; MetS. Metabolic syndrome; *Mttp*. Microsomal triglyceride transfer protein; *Myd 88*. Myeloid differentiation primary response 88; *Nfkb*. Nuclear factor kappa B; *Npc1l1*. Niemann-Pick C1-like 1; *Ocln*. Occludin; OxS. Oxidative stress; PAC. Proanthocyanidins; *Perk*. Protein kinase RNA-like ER kinase; *Pcsk9*. Proprotein convertase subtilisin 9; *Rxr*. Retinoid X receptor; SCFAs. Short-chain fatty acids; *Shp*. Small heterodimer partner; *Scarb 1*. Scavenger receptor class B, member 1; TC. Total cholesterol; TG. Triglycerides; TNFA. Tumor Necrosis Factor-alpha (protein); *Tnfa*. Tumor Necrosis Factor-alpha (gene); WAT. White adipose tissue; *Xbp1*. X-box-binding protein-1; ZO1. Zonula occludens-1.
