# Supplementary material for: Therapeutic Potential of Cranberry Proanthocyanidins in Addressing the Pathophysiology of Metabolic Syndrome: A Scrutiny of Select Mechanisms of Action
Source: Antioxidants (Basel). 2025 Feb 26;14(3):268. doi: 10.3390/antiox14030268 (PMC11939394; doi:10.3390/antiox14030268)
Supplement: Supplementary file 1 [file antioxidants-14-00268-s001.zip › antioxidants-3394700-supplementary.pdf]

SUPPLEMENTARY MATERIAL

TABLE S1. List of primers used for RT-qPCR analysis

	Primers
ZO1	Forward: TGCTGTTCAGCAGCTAAGGA
	Reverse: AGCTCTTGGGTCATGCACTT
Cldn1	Forward: TGGAAGATGATGAGGTGCAG
	Reverse: CCTGGCCAAATTCATACCTG
Ocln	Forward: GTTGATCCCCAGGAGGCTAT
	Reverse: GGGGGCTGTTCATCATAAAT
Cox2	Forward: GCTGTACAAGCAGTGGCAAA
	Reverse: CCCCAAAGATAGCATCTGGA
Nfkb	Forward: AGCTTCACTCGGAGACTGGA
	Reverse: ACGATTTTCAGGTTGGATGC
Ikb	Forward: TGGCCAGTGTAGGCAGTCTTG
	Reverse: GACACGTGTGGCCATTGTAG
Tnfa	Forward: GAACTGGCAGAAGAGGCACT
	Reverse: AGGGTCTGGGCCATAGAACT
Il6	Forward: GTTCTCTGGGAAATCGTGGA
	Reverse: TTCTGCAAGTGCATCAT
Grp78	Forward: TGCAGCAGGACATCAAGTTC
	Reverse: TACGCCTCAGCAGTCTCCTT
Grp94	Forward: GGCTCATCTCCCTAACTGATG
	Reverse: TGGTGCCGAGATTTTTAACC
ATF6	Forward: TGTCACTGGTCCTGGAAACA
	Reverse: TGAATGATGATGGCTTTTGC
Perk	Forward: ATCTGTTCTGCCTTGGGATG
	Reverse: CAAAGTGGCCAACACTGAAA
Ire1	Forward: CGCATCACCAAGTGGAAGTA
	Reverse: CCTTCCAGCAAAGGAAGAGT

Xbp1	Forward: GAGCAGCAAGTGGTGGATTT
	Reverse: CACCAGCCTTACTCCACTCC
Apob	Forward: TGAATGCACGGGCAATGA
	Reverse: GGCATTACTTGTTCCATGGTTCT
Mttp	Forward: ATGATCCTCTTGGCAGTGCTT
	Reverse: TGAGAGGCCAGTTGTGTGAC
Hmgcr	Forward: ATTCTGGCAGTCAGTGGGAACT
	Reverse: CCTCGTCCTTCGATCCAATTTA
Ldlr	Forward: GCTCCATAGGCTATCTGCTCTTCA
	Reverse: CTGCGGTCCAGGGTCATC
Pcsk9	Forward: GCACCAGACAGAGGAAGACC
	Reverse: GTGACCCTGCCCTCAATCT
Cd36	Forward: GCCAAGCTATTGCGACATGA
	Reverse: ATCTCAATGTCCGAGACTTTTCAAC
Npc1l1	Forward: CCACAGACCCTGTGGAACTG
	Reverse: GCTCGTCATGGAAAGCCTTT
Scarb1	Forward: TCAGAAGCTGTTCTTGGTCTGAAC
	Reverse: GTTCATGGGGATCCCAGAGA
Apoa1	Forward: CCACACCCTTCAGGATGAAAG
	Reverse: TGGCTCCCTGTCAGGAAGAC
Abca1	Forward: AGGGTTTCTTTGCTCAGATTGTC
	Reverse:TGCCAAAGGGTGGCACA
Fxr	Forward: GGCCTCTGGGTACCACTACA
	Reverse: ACATCCCCATCTCTTTGCAC
Shp	Forward: AGCTGGGTCCCAAGGAGTAT
	Reverse: CTTGAGGGTAGAGGCCATGA
Rxr	Forward: CTTTGACAGGGTGCTAACAGAGC
	Reverse: ACGCTTCTAGTGACGCATACACC
Cyp7a1	Forward: AGGACTTCACTCTACACC

	Reverse: TGGTCTTTGCTTTCCCACTT
Fgf15	Forward: GCTGGTCCCTATGTCTCCAA
	Reverse: CAGTCCATTTCCTCCCTGAA
Lbp	Forward: GATCACCGACAAGGGCCTG
	Reverse: GGCTATGAAACTCGTACTGCC
Myd88	Forward: GGCCTTGTTAGACCGTGAGG
	Reverse: GTGGGACACTGCTTTCCACT
Cd14	Forward: CTGATCTCAGCCCTCTGTCC
	Reverse: GCTTCAGCCCAGTGAAAGAC
Actin	Forward: GACAGGATGCAGAAGGAGATTACTG
	Reverse: CCACCGATCCACACAGAGTACTT

TABLE S2. Histology score of liver disease

Index<br><br>Scoring	Lobular inflammation (0-3)	Steatosis<br><br>(0-3)	Hepatocellular balloning<br>(0-2)	Total score<br><br>(0-8)
**Chow**	0.50+0.20	0+0	0+0	0.50+0.20
**HFHF**	1.25+0.25[&]	2.25+0.25***	0.75+0.25*	4.25+0.25***
**HFHF+PAC**	1.00+0.41	1.50+0.50	0+0#	2.5+0.65#

Results are presented as mean ± SEM for n = 6-8 mice/group. & $p < 0.1$, * $p < 0.05$, *** $p < 0.001$ vs. Chow; # $p < 0.05$ vs. HFHF mice.

TABLE S3. Concentrations of fecal SCFA

Time (week)	SCFA	Chow			HFHF			HFHF+PAC		
T=0	Acetic Acid	17.672	±	1.654	21.118	±	2.410	16.678	±	3.311
	Propionic Acid	4.968	±	1.016	5.191	±	0.703	3.743	±	0.939
	Isobutyric Acid	0.114	±	0.043	0.068	±	0.033	0.066	±	0.032
	Butyric Acid	4.125	±	0.658	7.115	±	1.511	2.481	±	0.382
	2-Methyl Butyric Acid	0.073	±	0.020	0.067	±	0.022	0.018	±	0.012
	Isovaleric Acid	0.116	±	0.023	0.115	±	0.015	0.072	±	0.014
	Valeric Acid	0.349	±	0.043	0.378	±	0.071	0.151	±	0.033
	2-Methyl Valeric Acid	0.005	±	0.005	0.004	±	0.004	0.000	±	0.000
	Isocaproic Acid	0.000	±	0.000	0.000	±	0.000	0.000	±	0.000
	Caproic Acid	0.000	±	0.000	0.000	±	0.000	0.000	±	0.000
	**Total**	**27.423**	**±**	**2.538**	**34.056**	**±**	**3.989**	**23.210**	**±**	**4.552**
	**Subtotal (a+p+b)**	**26.766**	**±**	**2.426**	**33.424**	**±**	**3.938**	**22.902**	**±**	**4.531**
	**Subtotal (other)**	**0.657**	**±**	**0.124**	**0.632**	**±**	**0.097**	**0.308**	**±**	**0.082**
T=11	Acetic Acid	30.302	±	7.342	11.424	±	1.621*	22.726	±	6.162
	Propionic Acid	4.888	±	0.477	1.785	±	0.371***	5.157	±	1.415#
	Isobutyric Acid	0.179	±	0.030	0.381	±	0.084	0.767	±	0.204
	Butyric Acid	8.742	±	3.497	0.863	±	0.157*	1.381	±	0.317
	2-Methyl Butyric Acid	0.122	±	0.030	0.212	±	0.045	0.474	±	0.124#
	Isovaleric Acid	0.172	±	0.029	0.267	±	0.061	0.397	±	0.099
	Valeric Acid	0.493	±	0.129	0.345	±	0.084	0.510	±	0.148

Time (week)	SCFA	Chow	HFHF	HFHF+PAC
	2-Methyl Valeric Acid	0.005 ± 0.005	0.044 ± 0.044	0.000 ± 0.000
	Isocaproic Acid	0.000 ± 0.000	0.041 ± 0.041	0.000 ± 0.000
	Caproic Acid	0.000 ± 0.000	0.038 ± 0.038	0.000 ± 0.000
	**Total**	**44.829 ± 10.893**	**15.401 ± 2.338***	**31.235 ± 8.346**
	**Subtotal (a+p+b)**	**43.933 ± 10.725**	**14.071 ± 2.036***	**29.265 ± 7.798**
	**Subtotal (other)**	**0.896 ± 0.198**	**1.330 ± 0.361**	**1.971 ± 0.569**
TΔ	Acetic Acid	12.630 ± 8.026	-9.694 ± 3.340*	6.048 ± 8.118
	Propionic Acid	-0.080 ± 1.207	-3.406 ± 0.794*	1.414 ± 2.087#
	Isobutyric Acid	0.027 ± 0.052	0.314 ± 0.095*	0.690 ± 0.229
	Butyric Acid	4.617 ± 3.441	-6.253 ± 1.440*	-1.100 ± 0.544##
	2-Methyl Butyric Acid	0.021 ± 0.044	0.146 ± 0.053	0.452 ± 0.130#
	Isovaleric Acid	0.056 ± 0.037	0.152 ± 0.065	0.325 ± 0.105
	Valeric Acid	0.144 ± 0.140	-0.033 ± 0.109	0.359 ± 0.165
	2-Methyl Valeric Acid	0.000 ± 0.008	0.040 ± 0.045	0.000 ± 0.000
	Isocaproic Acid	0.000 ± 0.000	0.041 ± 0.041	0.000 ± 0.000
	Caproic Acid	0.000 ± 0.000	0.038 ± 0.038	0.000 ± 0.000
	**Total**	**17.406 ± 11.817**	**-18.655 ± 4.960***	**8.026 ± 11.162#**
	**Subtotal (a+p+b)**	**17.167 ± 11.607**	**-19.353 ± 4.765***	**6.363 ± 10.615#**
	**Subtotal (other)**	**0.239 ± 0.238**	**0.698 ± 0.378**	**1.663 ± 0.606**

SCFA: Short chain fatty acid. Subtotal (a+p+b) reflects the total SCFA concentrations of acetic acid, propionic acid and butyric acid, whereas the subtotal (other) was obtained following the subtraction of the total and subtotal (a+p+b) concentrations. Results are presented as mean ± SEM for n = 6-8 mice/group. * $p < 0.05$, *** $p < 0.001$ vs. chows; # $p < 0.05$, ## $p < 0.01$ vs. HFHF mice.

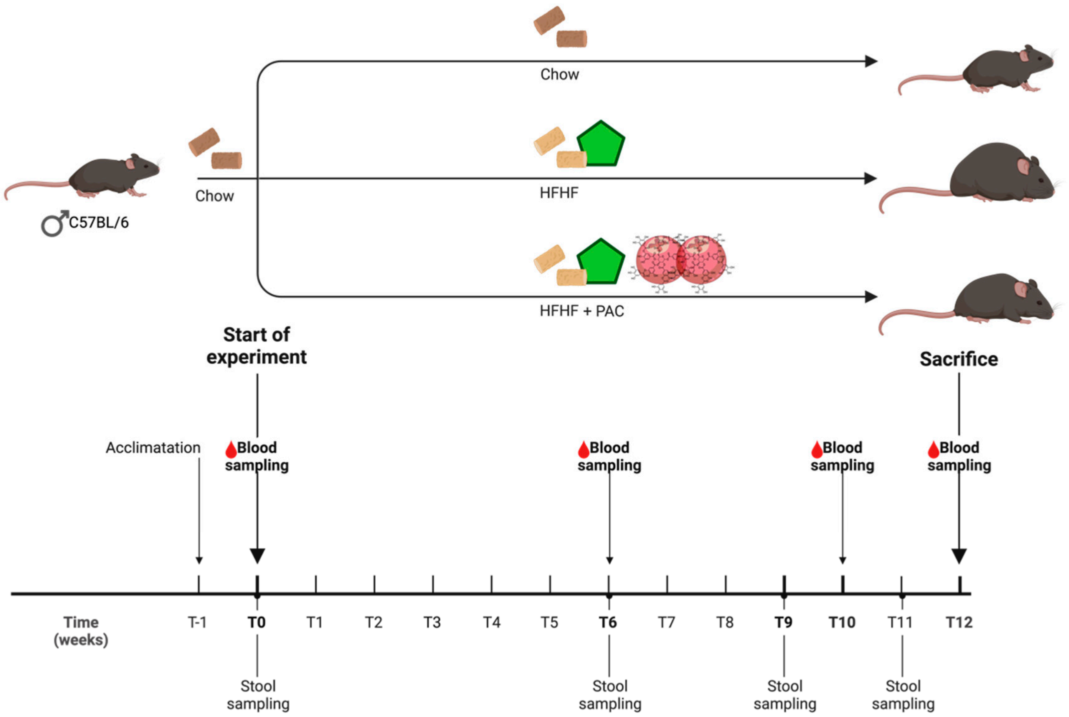

Figure S1. Experimental design. Eight-week-old C57BL/6J male mice (Charles River; Montreal, QC, Canada) were housed in a controlled environment (22±1°C; 12/12 hours light dark) with free access to food and drinking water. Following one week-acclimation on a standard diet, mice were fed either a Chow diet or a high-fat, high fructose (HFHF)-regimen, consisting of a combination of a high-fat, high-sucrose diet as well as 30% fructose in drinking water, with daily PAC doses of 200 mg/kg body weight by gavage (HFHF+PAC). The Chow- and HFHF-nourished mice received the vehicle (water) by gavage in order to mimic the conditions in the HFHF+PAC group. Body weight gain and food intake were assessed twice a week. Stool samples were collected at weeks 0, 6, 9 and 11. Blood was collected from the tails of mice at weeks 0, 6 and 10 following a 6-hour fast.

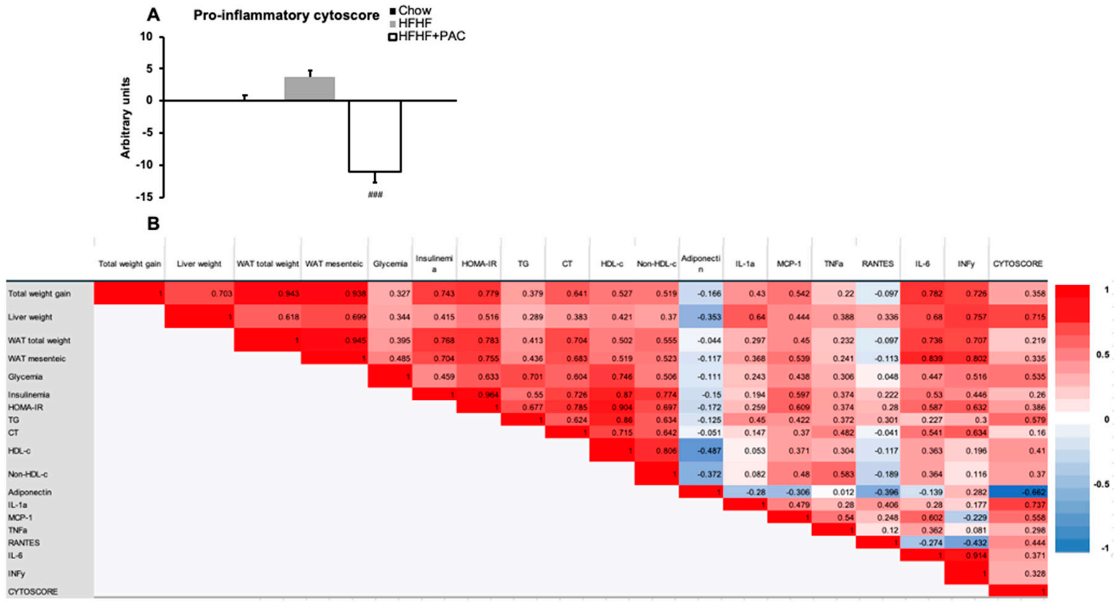

Figure S2. Effect of PAC on inflammatory circulating biomarkers and relationship with anthropometric and metabolic parameters. Mice were fed either a standard chow diet, a high-fat, high-fructose diet (HFHF) or HFHF+PAC for 12 weeks. Plasma samples were submitted to multiplex analysis for quantification of circulating markers G-CSF, IFNγ, IL-1α, IL-1β, IL-2, IL-4, IL-5, IL-6, IL, 7, IL-9, IL-10, IL-12p40, IL-12p70, IL-13, IL-15, IL-17, KC, LF, LIX, MCP-1, M-CSF, MIG, MIP-1α, MIP-1β, MIP-2, RANTES, TNFα and VEGF. Linear combination of all 31 analytes into a (**A**) inflammatory cytoscore for all three groups; data are expressed as mean ± SEM relative to chow for n = 6-8 mice/group. ### p < 0.001 vs. HFHF mice. (**B**) Combination matrices with colors representing the Spearman R value (blue: negative association -1; red positive association 1) between all multiplex analytes and selected markers : mice body weight (BW), triglyceridemia (TG), total cholesterolemia (TC), adiponectin and homeostatic model assessment of insulin resistance (HOMA-IR). Significant p-values (p<0.05) are indicated in highlighted box.

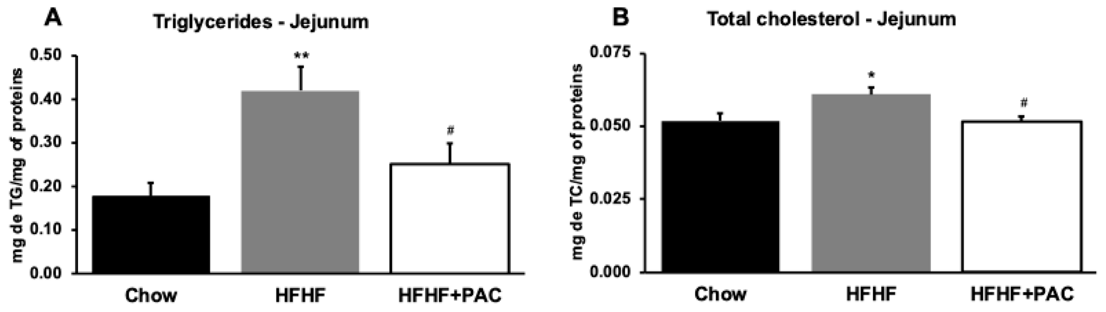

Figure S3. Effect of PAC on gut lipid accumulation. (**A**) Triglycerides and (**B**) total cholesterol content was quantified in jejunum tissues as described in Materials and Methods section. Results are presented as mean ± SEM for n = 12 mice/group. * $p < 0.05$, ** $p < 0.01$ vs. chows; # $p < 0.05$ vs. HFHF mice.

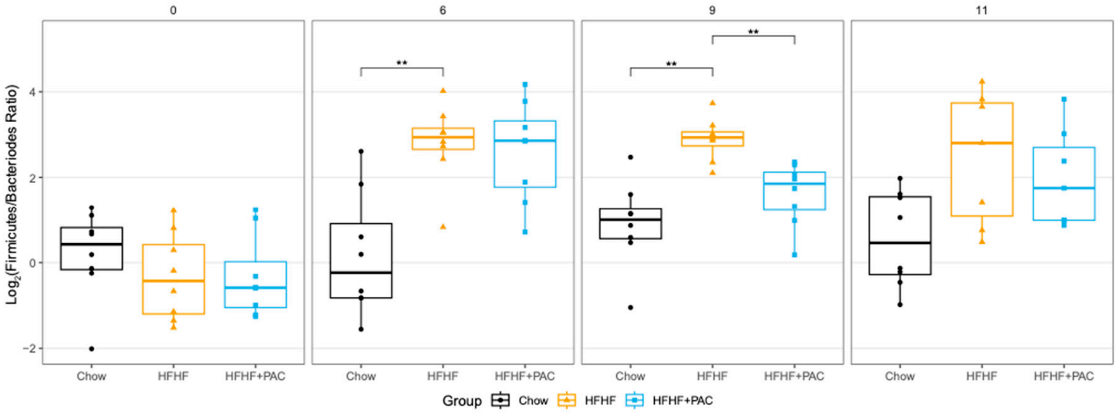

Figure S4. Firmicutes/Bacteroidetes ratio over time (weeks). ** $p < 0.01$.

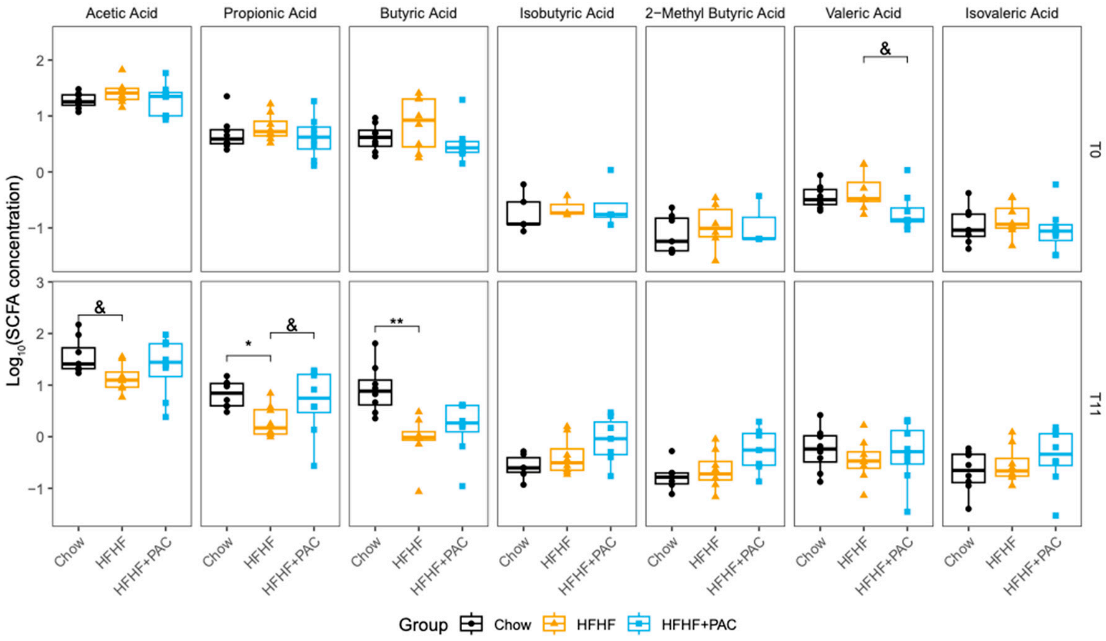

Figure S5. Levels of measured SCFAs at for each group at T0 or after 11 weeks of the dietary intervention. Boxplots show the interquartile range with the median represented as a line. & $p < 0.1$, * $p < 0.05$.
